# Estimating Risks of Inapparent Avian Exposure for Human Infection: Avian Influenza Virus A (H7N9) in Zhejiang Province, China

**DOI:** 10.1038/srep40016

**Published:** 2017-01-05

**Authors:** Erjia Ge, Renjie Zhang, Dengkui Li, Xiaolin Wei, Xiaomeng Wang, Poh-Chin Lai

**Affiliations:** 1 Division of Epidemiology, Dalla Lana School of Public Health, University of Toronto, Toronto, Canada; 2 Zhejiang Provincial Center for Disease Prevention & Control, Hangzhou, P.R. China; 3 School of Mathematics & Statistics, Xi’an Jiaotong University, Xi’an, P.R. China; 4 Division of Clinical Public Health and Institute of Health Policy, Management and Evaluation, Dalla Lana School of Public Health, University of Toronto, Toronto, Canada; 5 Department of Geography, The University of Hong Kong, Hong Kong SAR

## Abstract

Inapparent avian exposure was suspected for the sporadic infection of avian influenza A(H7N9) occurring in China. This type of exposure is usually unnoticed and difficult to model and measure. Infected poultry with avian influenza H7N9 virus typically remains asymptomatic, which may facilitate infection through inapparent poultry/bird exposure, especially in a country with widespread practice of backyard poultry. The present study proposed a novel approach that integrated ecological and case-control methods to quantify the risk of inapparent avian exposure on human H7N9 infection. Significant associations of the infection with chicken and goose densities, but not with duck density, were identified after adjusting for spatial clustering effects of the H7N9 cases across multiple geographic scales of neighborhood, community, district and city levels. These exposure risks varied geographically in association with proximity to rivers and lakes that were also proxies for inapparent exposure to avian-related environment. Males, elderly people, and farmers were high-risk subgroups for the virus infection. These findings enable health officials to target educational programs and awareness training in specific locations to reduce the risks of inapparent exposure.

Avian influenza A(H7N9) virus, detected strictly in birds in the past, finally spread to humans and animals in China in February/March of 2013^1^. The virus is low pathogenic for avian hosts, resulting in asymptomatic or mild avian diseases^2^. This asymptomatic infection of poultry may cause a ‘silent’ poultry-to-human transmission through direct contact with or exposure to seemingly healthy but infected birds^3^. The number of laboratory-confirmed H7N9 cases in China reached 511 within a span of just 1.5 months; with 209 deaths from 12 provinces and two municipalities^4^. Although incorrectly lumped with other avian influenza viruses at the outset, the H7N9 virus originating from southeastern coast of China continued its spread to other regions^5^,^6^. It was soon affirmed that the H7N9 virus is a global issue in public health^7^ because the virus has obtained strong abilities of jumping from its avian origins to mammals to cause severe illnesses and even deaths in human.

Direct poultry contact and exposures to poultry in live-poultry markets (LPMs) are potential sources of H7N9 infection^8^,^9^,^10^,^11^. LPMs have been reported as a main source of H7N9 transmission by way of human-poultry contact and avian-related environmental exposures^12^,^13^,^14^. The closures of LPMs appeared to decrease the risks of H7N9 infection in many Chinese cities such as Shanghai, Hangzhou, Huzhou and Nanjing^15^. However, the intervention was not always effective as demonstrated in Hangzhou and other counties of the Zhejiang province where new cases occurred after LPMs had been permanently closed in the winters of 2014 and 2015^16^,^17^,^18^,^19^. The H7N9 virus continued to spread not only around LPMs, backyards, and small-scale poultry farms but also in natural environmental settings that were more difficult to detect^20^. Inapparent avian exposure might have contributed to the sporadic re-emergence of the H7N9 infection in Hangzhou even though all the LPMs were permanently shut down in this city^21^,^22^.

Inapparent poultry exposures are very common in people’s daily lives in China, although this type of exposure is difficult to be noticed, identified, and measured. The usual way to record such exposure was through recall interviews and questionnaire surveys. However, the ‘inapparent’ nature also means that an interviewee might not even be aware of the presence of poultry or wild birds in his/her surroundings to provide an accurate account. Measurement irregularities also occurred because of perceptual differences in ordinal frequency (often, sometimes, seldom, never) or intensity (none, mild, moderate, severe). Poultry and LPM densities have been used as proxies for estimating the risks of outdoor environmental exposure to poultry^23^. In these studies, areal units (such as 1 × 1 km^2^ pixel) without H7N9 cases were randomly selected to generate pseudo-absence data on infection. However, the risk of poultry-related exposure might be underestimated as some of the selected areas of pseudo-absence might have locally high poultry densities relative to their human population.

The objective of this study was to examine the effects of inapparent avian exposure on human H7N9 infection. Spatial/ecological and case-control studies were integrated to estimate the exposure risks at the individual level after adjusting for spatial and personal demographic factors across multiple spatial scales. The novelty of this case-control study was the use of two different diseases of comparable but unrelated infectious etiology as cases and controls. Due to limited availability of data for noncases, this study employed H7N9 as cases and tuberculosis (TB) as controls. Specifically, the study made an attempt to answer the following questions: 1) Are poultry densities, species, and distances/proximity to inland waters (i.e., rivers and lakes) associated with human H7N9 infection and what are the relative contributions of each variable to the occurrence of human H7N9 infection? 2) Do these variables vary geographically and exert interactive impacts to increase the risks of infection? and 3) Who are the target high-risk populations: male vs. female, adult vs. elderly, or farmer vs. worker vs. other occupation subgroups?

## Results

Table 1 summarized characteristics of the cases and controls matched by age, gender, and occupation. There were no significant differences between the groups with respect to age (*p* = 0.831), gender (*p* = 0.986), and occupation (*p* = 0.106). Over 97% of cases occurred in adults aged 15 years or older, with 51% belonging to the working-age population (aged 15–60). The number of male cases was 93 which almost doubled the number of females. The proportion of farmer infection was around 41% and four times higher than that of the worker subgroup in non-agricultural occupations. The ‘other’ subgroup included agricultural workers in forestry, animal husbandry, fishery, agricultural machinery, and hunting, which accounted for 47.9% of all infected cases.

The average shortest distances to inland waters were 25.7 kilometers (*km*) for rivers and 100.9 *km* for lakes for the case group and 39.4 *km* and 183.8 *km* respectively for the control group. Areas surrounding the cases had consistently higher poultry densities than those near the controls. Statistically significant case-control differences (*p* < 0.05) were found in the average chicken and goose densities across multiple spatial scales: neighborhood (<1 *km*), community (1–3 *km*), district (3–5 *km*), and city (5–8 *km*). Duck density did not show such significant case-control differences at the first two spatial scales. The above variables were further categorized into ordinal groups to examine their association with risks of human H7N9 infection (Supplementary Table S1).

Table 2 summarized the interaction effects between inland waters and poultry densities at the neighborhood level. The effects of inapparent poultry exposure consistently increased with proximity to inland waters at community, district, and city levels (Supplementary Table S2). The *q-*statistics of the shortest distances to rivers and lakes were 0.014 and 0.050 respectively using the geographical detector method^24^. The spatial stratified heterogeneity analysis^25^ indicated significant association (*p *< 0.05) between the occurrences of H7N9 infection and the shortest distances to inland waters. For the poultry densities, the *q* statistics were 0.032 to 0.058 for chicken, 0.031 to 0.059 for goose, and 0.005 to 0.030 for duck. Chicken and goose showed significant association with H7N9 infection at the four spatial scales while duck did not. The infection risks were further elevated by interaction effects between inland waters and poultry densities.

The stratified/conditional logistic regression analyses^26^ indicated that the human H7N9 infection was significantly associated with chicken and goose densities after adjusting for clusters of infection cases across the four spatial scales (Table 3 and Supplementary Table S3). The matched odd ratios (mOR) of H7N9 infection per unit increase in the chicken density were 1.6 (95% CI, 1.0, 2.9), 2.9 (1.4, 6.2), 3.5 (1.6, 7.7), and 3.3 (1.3, 7.3) at neighbourhood, community, district, and city levels respectively. Goose density with mOR values of 2.8 (1.2, 6.9), 5.0 (1.8, 3.5), 5.9 (2.0, 17.7), and 5.8 (1.9, 17.9) across the four spatial scales had stronger associations with H7N9 infection compared with the other two species. Males, elderly people, and farmers with comparatively high mOR values were high-risk population subgroups susceptible to H7N9 infection in conjunction with increases in chicken and goose densities. The ‘Other’ occupation subgroup had noticeably high risks of H7N9 infection in association with chicken densities. This population subgroup which embraced ‘unclassified’ workers, including poultry handlers and those working in restaurants not discernable through the census, might have confounded the results. Duck density did not show significant association with human H7N9 infection among the various population subgroups.

Table 4 showed results of the multivariate logistic regression. Chicken and goose demonstrated significant association with H7N9 infection at the community and district levels, although the risks (i.e., mOR) were reduced by incorporating proximity to inland waters into the analyses. The relative risks compared with the single poultry model (Table 3 and Supplementary Table S3) reduced by 0.25 (1.6 vs. 1.35), 0.94 (2.9 vs. 1.96), 1.39 (3.5 vs. 2.11), and 1.27 (3.3 vs. 2.03) for chicken densities at the neighborhood, community, district, and city levels respectively. The mORs of goose density (ranging from 1.78 to 2.87 in Table 4) were also modified by incorporating proximity to inland waters (0.996 to 0.997 in Table 4), both of which were lower than 2.8 to 5.9 estimated by the single poultry models (Table 3 and Supplementary Table S3). The shortest distances to inland waters (reporting mORs of <1.0 in Table 4) exhibited significant negative association with H7N9 infection. That is to say the closer distances from rivers and lakes, the greater the risks of H7N9 virus infection. Indeed, staying away from inland waters would minimize inapparent poultry exposure and the likelihood of human H7N9 infection. Disease clusters also implicated increased risks of inapparent avian exposure for human infection, which can be a confounding factor for this study. Significant associations between disease clusters and human H7N9 infection were noted across all four spatial scales but neighborhood-level disease clusters markedly increased the odds of human infection.

## Discussion

This is a novel study that integrates epidemiology and health geography to quantify the effects of inapparent exposure to poultry and proximity to inland waters on human H7N9 infection through a set of individual-level cases (H7N9) and their matched controls (TB). Spatial stratified heterogeneity analyses were applied to examine the connections between poultry densities and the shortest distances to inland waters, both were proxies for inapparent avian exposure and their associations with the virus infection. Spatial clusters, which could be a confounding factor in the analyses, were estimated to adjust for potential, albeit very limited^27^, human-to-human transmissions. This case-control study on avian influenza viruses, compared with traditional ecological studies^28^, extends from the present day focus on population/group level factors in infectious-disease epidemiology to an individual-level analysis. At the same time, the method adds flexibility to adjust for spatial and personal confounding factors and accommodate ecological variability.

### Inapparent avian exposure vs. exposure to live poultry markets (LPMs)

Inapparent avian exposure is a risk factor for human H7N9 infection although it is not directly observable or easily noticeable through visible contacts with poultry in the fields or in LPMs. This study reported that the adverse effects of inapparent avian exposure on human H7N9 infection varied by poultry species, poultry densities, and proximity to inland waters. These findings have serious public health implications for human H7N9 prevention and control. A lower infection risk at the neighborhood scale (≤1 *km*), compared to higher risks at the community, district or city levels (i.e., 1–3 *km*, 3–5 *km* or 5–8 *km*, see Table 2) implied that people were more likely infected through inapparent exposure to sick birds or poultry-related environment during outdoor activities beyond their local neighborhoods. It was also possible that those living in close proximity to poultry farms would consciously take safety measures or stay away from the farms if not engaged in the poultry business. By the same token, people tended to let their guards down when they were further away from poultry farms and not knowing that they were being exposed. Inapparent avian exposure usually goes unnoticed because the effects of such exposure are rarely immediate and the symptoms are not fully recognized. For example, studies have shown that the transport of livestock and poultry between farms and markets occurred along the same road networks used for recreation and commuting to work^11^. No protective measures against potential infection would take place in these situations simply because people were unaware of their risks of avian exposure.

It has been demonstrated in many ecological studies that avian infection around the LPMs decreased with increasing distance from the markets^28^,^29^. However, inapparent avian exposure is difficult to model as it is dynamic and spatially heterogeneous. This study managed to model inapparent avian exposure by including not only proximity to waterbodies but also the spatial distribution of poultry species and flock densities with adjustments for disease clustering effects. The results indicated that mere closures of LPMs would not be effective in controlling virus infection and its spread, which was consistent with previous findings^18^,^19^.

### Poultry species and H7N9 infection

There were few ecological and epidemiological studies about selected species of birds most likely to be infected and shed the virus to infect human. This study suggested human avian infection was significantly associated with chicken and goose densities (but not duck) within a distance of 8 *km* (Table 2). Goose exhibited the highest risks among the three poultry species for human avian infection with age, gender, and occupation adjusted (Table 3). These risks were verified statistically and consistent with findings by Pantin-Jackwood and his colleagues^30^ that concluded quail and chicken were susceptible to infection and had important roles in virus transmission to human whereas other species such as duck and pigeon played a lesser role. Our results also showed that inapparent exposure to chicken or chicken-related environment more than other species was more harmful to the elderly (Table 3 and Supplementary Table S3). Further studies are needed to explain why goose had the largest effects on human H7N9 infection.

### Risks of H7N9 infection stratified by age, gender, and occupation subgroups

Individual characteristics also affect susceptibility to infection^31^. This study found that men, elderly people ≥60, and farmers were more likely affected through inapparent exposure to chicken and goose (Table 3 and Supplementary Table S3). It is customary that adults aged above 60 in China were retired and took responsibility for most household tasks^11^. They traveled frequently to wet markets and thus had a higher probability of exposure to avian or avian-related environment than younger adults working in indoor office environments. The higher risks of the elderly could be attributed not only to personal medical conditions^8^,^32^ but also their daily behaviors of visiting wet markets, as well as conditions of their living environment^33^,^34^.

This study identified the male population, accounting for 65% of all infection cases, as a high-risk subgroup although a previous study in Zhejiang did not find a substantial difference in poultry exposure between the two genders^35^. A higher risk in male adults could be related to their behaviors and bird-related hobbies. Oriental males were more likely to visit wild-bird markets and keep pet birds at home. Infected birds carrying avian influenza viruses might go undetected before they were sold. Cockfights, which is a cultural activity favored by men in many tropical and subtropical Asian countries, might also play a role in transmitting avian influenza viruses to human^36^. These behaviors would have increased the potential of inapparent exposure to infected avian and avian-related environment.

The farmer subgroup reported significant and higher risks of infection with the H7N9 virus through chicken and goose across a large distance, as indicated by the mORs (Table 3 and Supplementary Table S3). Farmers working in rural farms had higher risks of inapparent avian exposure because of increased likelihood of their domestic poultry to be exposed to viruses carried by wild birds^37^. The ‘other’ subgroup also demonstrated significantly high risks of H7N9 infection at the neighborhood level. This subgroup included wet market pedals, poultry handlers, and those working in restaurants. These workers were likely subject to intense inapparent exposure to infected droppings or to environments contaminated by infected poultry. Unlike the avian influenza H5N1 virus, poultry infected with the H7N9 virus showed little to no symptoms of illness which increased the opportunity for farmers to become infected. Effect differences between the two subgroups could be attributable to exposure intensity, spaciousness, and time scales that could not be explained simply by the number of poultry or species.

### Limitations and Conclusions

This study had a few limitations. Firstly, serum specimens were not collected from the controls to determine seropositivity to H7N9 virus. Although no known cases of simultaneous infection of H7N9 and TB had been reported in the province of Zhejiang, the statistical power of estimating the effects of inapparent exposure would be reduced when some of the controls were confirmed seropositive to avian influenza virus. Secondly, although disease clusters were estimated to correct for the potentials of spatial transmission among people (Table 4), our data offer no information on individual habits (smoking, drinking, diet, etc.), health conditions (chronic diseases, obesity, hypertension, etc), and socio-economic status (education attainment, household income, etc.). These confounders could have influenced our results and their absence prevented us from conducting more detailed analyses. Finally, this cross-sectional study did not allow for examination of temporal or seasonal effects of inapparent avian exposure. It is generally known that a large number of live poultry is transported between rural poultry farms and wet markets in urban areas during the days of Chinese spring festival in January or February of each year^17^. This time period of festivities would be associated with increased exposure to infected poultry through direct or indirect (or inapparent) contact with avian influenza viruses. Longitudinal surveys should be conducted in future studies to examine seasonal variation of exposure effects and risks of human infection with the viruses.

In conclusion, this study operationalized a means to account for inapparent avian exposure and demonstrated the risk trends of H7N9 virus in human infection associated with poultry density, species, and avian-related environment such as proximity to inland waters. The risks vary in population by age, gender, and occupation. Thus, health education to bring awareness of this risk is needed to protect the public from inapparent exposure to avian influenza viruses. The research findings can also gauge the development of protective measures and strategies in accordance with population characteristics and the spatial separation of poultry farms.

## Materials and Methods

### Study area

**Figure 1:**
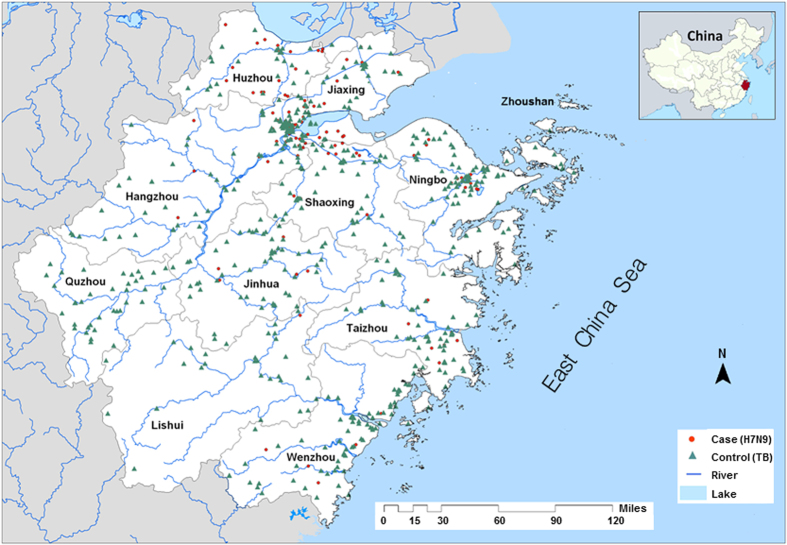
Study area and point map of disease locations. Zhejiang is a province on the east coast of China. Two types of diseases were considered in this case-control study: 142 human H7N9 cases (red dots) and four times the case number of tuberculosis controls (green triangles). Figure was compiled by author using the software “ArcGIS, [v.9.3], (https://www.arcgis.com/)” and the following digital spatial data: (1) boundary map of the Zhejiang township from the National Data Sharing Infrastructure of Earth System Science (Geodata Centre at http://www.geodata.cn) and (2) disease data about tuberculosis and avian influenza H7N9 between March 2013 and July 2014 from the Zhejiang Provincial Center for Disease Control and Prevention (http://www.cdc.zj.cn/bornwcms/ Templets/new_cdc/english.htm). We geocoded home addresses of individuals and plotted their locations using ArcGIS v.9.3. The figure was touched up using Windows Microsoft Paint tool.

Zhejiang, located south of the Yangtze River Delta, is a province on the east coast of China (Fig. 1). It had the largest number of confirmed human H7N9 infection between 2013 and 2014^38^. But unlike other provinces of Mainland China, Zhejiang had 66.7% cases occurring in the rural suburbs^39^ where inapparent avian exposure most likely happened in backyards, poultry farms, and environments contaminated by infected birds^19^,^38^.

### Data

The study employed two types of disease data with spatial locations: H7N9 (cases) and TB (control). Other data included the spatial distribution of three poultry species (i.e., chicken, duck, and goose) and their numbers, as well as locations of inland waters (i.e., rivers and lakes).

Human H7N9 infection was defined according to the diagnostic and treatment program issued by the National Health and Family Planning Commission of the People’s Republic of China^40^. A total of 142 laboratory-confirmed H7N9 cases were obtained from the Zhejiang Provincial Centre for Disease Prevention and Control (Zhejiang CDC) for the period between March 2013 and July 2014. Each case contains information on patient ID, age, gender, occupation, home address, and dates of symptom onset, hospital admission, and death. The home address records the structural hierarchy of administrative divisions of the People’s Republic of China comprising of street number, street name, district name, and city name. The confirmed cases were mapped by their home addresses using geocoding tools in ArcGIS. Figure 1 shows the spatial distribution of H7N9 cases (as red solid circles) within the province of Zhejiang.

TB data were obtained as controls from the Zhejiang CDC for the same time period covered by H7N9 cases. The controls were randomly selected but matched in proportions by age (<15, 15–59, and ≥60 years old), gender (male and female), and occupation (farmer, worker, and others). The number of TB controls was four-times the size of H7N9 cases. The controls were also geocoded by their nominal addresses and the spatial distribution (as green solid triangles) was displayed in Fig. 1.

Poultry densities (chicken, duck, and goose) were used as proxies for risks of inapparent avian exposure. Digital maps of poultry density in high resolution format of 1×1 *km*^2^ were obtained from a previous study^41^. Data on the distribution of inland waters, including rivers and lakes, were obtained from the National Data Sharing Infrastructure of Earth Systems Science^42^. This study assumed that the closer is an area to a waterbody, the higher is the probability of inapparent exposure to birds or poultry.

### Study design

This is a spatial case-control study that employed spatial point and spatial stratified heterogeneity^25^ analyses. For a set of human H7N9 infection cases reported between March 2013 and July 2014, a collection of noncases (i.e., controls) representing an independent random sample of subjects free of H7N9 within the same time period was matched to the set of H7N9 cases in similar proportions by demographic traits such as age, gender, or occupation. Due to limited availability of point data for noncases, this study employed TB cases as a control bearing no relation to H7N9 and avian-related environment^8^,^43^. This study assumed exposure among cases and controls was different to examine impacts of inapparent exposure to avian-related environments on H7N9 infection.

Inapparent avian exposure was modeled using the spatial distribution of poultry species, their respective densities, and the shortest distances to inland waters. Multiscale spatial analyses (including spatial stratified heterogeneity analysis and spatial cluster analysis) were applied to estimate inapparent avian exposure across four spatial scales. Since 52% of the Chinese population commuted between 2 to 8 kilometers every day^44^, the four spatial scales were set at less than 1 km, 1–3 km, 3–5 km, and 5–8 km, which correspond to successive distance increments from one geographic unit to the next, viz. neighborhood → community → district → city. Within each spatial scale, average poultry densities and the shortest distances to inland waters were calculated for each case and control using ArcGIS 9.3 (www.arcgis.com) and the Matlab v.2010b mapping toolbox.

### Statistical analyses

The Wilcoxon rank sum statistics was used to examine the distribution of disease between groups of individuals with different demographic characteristics (i.e., age, gender, and occupation) for the case and control groups. *P*-values were generated to determine statistical significance.

### Spatial stratified heterogeneity analyses

The geographical detector method^24^ is a spatial analysis method for measuring spatial stratified heterogeneity^25^. It was applied to examine whether multiple variables (i.e., poultry densities and distances to inland waters) independently or dependently affect H7N9 occurrences. The two variables were divided into five ordinal categories using Jenk’s optimization^45^ data classification that reduces the within group variance while maximizes the between group variance. Here, distances were classed into five ordinal categories of very near, near, average, far, and very far whereas poultry densities into five classes of very low, low, average, high, and very high (Supplementary Table S1). The effects of the two influencing variables on H7N9 occurrences may be independent or dependent. The effects on H7N9 occurrences may be stronger or weaker after interaction (Table 2 and Supplementary Table S2).

### Stratified/conditional logistic regression analyses

Diseases can cluster spatially, temporally, or spatiotemporally to potentially cause spatial confounding. Stratified/conditional logistic regression analyses were applied to model the risks of human H7N9 infection associated with average poultry densities and proximity to inland waters after correcting for effects of disease clustering. The co-effects of inapparent exposure to different poultry species and proximity to inland waters were also modeled using multivariate logistic regression and adjusting for disease clustering. The local *K* function^26^, a widely-used measure of spatial clustering of points, was used to detect and identify the degree of clustering for cases and controls (Tables 3 and 4, and Supplementary Table S3).

Subgroup analyses were also conducted to model exposure risks by gender, age, and occupation groups. Children under 15 years of age were excluded from the study due to low counts. The modified effects of inapparent exposure to poultry on the risks of H7N9 infection were examined in conjunction with the shortest distances to inland waters using multivariate logistic regression after adjusting for disease clustering.

All of the above analyses were conducted across four spatial scales. The study employed the Epi package in R v.2.10 (R Development Core Team, Vienna, Austria) to carry out the case-control analyses using stratified/conditional and multivariable logistic regression models. The results were reported as mORs and their corresponding 95% confidence intervals.

## Additional Information

**How to cite this article:** Ge, E. *et al*. Estimating Risks of Inapparent Avian Exposure for Human Infection: Avian Influenza Virus A (H7N9) in Zhejiang Province, China. *Sci. Rep.* **7**, 40016; doi: 10.1038/srep40016 (2017).

**Publisher's note:** Springer Nature remains neutral with regard to jurisdictional claims in published maps and institutional affiliations.

## Supplementary Material

Supplementary Information

## Figures and Tables

**Table 1:** Statistical summary of H7N9 cases and matched controls by age, gender, and occupation.

Variables		Case (n = 142) Number (%)	Control (n = 559) Number (%)	*p*-value
Age (years old)	Children (≤15)	3 (2.11)	17 (3.04)	0.831
	Adult (16–60)	73 (51.41)	281 (50.27)	
	Elderly (≥60)	66 (46.48)	261 (46.69)	
Gender	Male	93 (65.49)	369 (66.01)	0.986
	Female	49 (34.41)	190 (33.99)	
Occupation	Farmer	59 (41.55)	235 (42.04)	0.106
	Worker	15 (10.56)	96 (17.17)	
	Others	68 (47.89)	228 (40.79)	

**Table 2:** Interaction between shortest distances to inland waters and poultry densities in contributing to H7N9 infection at the neighborhood level.

Interaction detector C = A∩B		Linear combination A+B	Graphical representation	Interpretation
river∩chicken = 0.076	>	0.046 = river(0.014)+chicken(0.032)	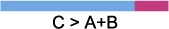	⇑
river∩goose = 0.063	>	0.045 = river(0.014)+goose(0.031)	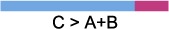	⇑
river∩duck = 0.050	>	0.019 = river(0.014)+duck(0.005)	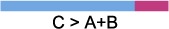	⇑
lake∩chicken = 0.086	>	0.082 = lake(0.050)+chicken(0.032)	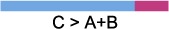	⇑
lake∩goose = 0.076	<	0.081 = lake(0.050)+goose(0.031)	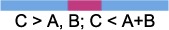	↑
lake∩duck = 0.071	>	0.055 = lake(0.050)+duck(0.005)	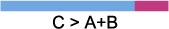	⇑

Note: A and B indicate inland waters and poultry densities respectively; A ⇑ B denotes nonlinear enhancement of A and B when C > A+B; A ↑ B denotes A and B enhance each other when C > A, B.

**Table 3:** Estimated risks (matched Odds Ratio; 95% Confidence Intervals) of H7N9 infection associated with poultry species and densities at the neighborhood level.

Variable	All population	Gender		Age (years old)		Occupation		
		Male	Female	15–59	≥60	Farmer	Worker	Others
	142 cases	93 cases	49 cases	73 cases	66 cases	59 cases	15 cases	68 cases
	599 controls	369 controls	190 controls	281 controls	261 controls	235 controls	96 controls	228 controls
Chicken	1.6(1.0,2.9)	2.5(1.1,6.6)	1.1(0.6,2.3)	1.6(1.0,3.6)	1.8(1.0,5.5)	1.7(1.0,4.1)	1.6(0.4,25.5)	2.1(1.0,7.3)
Cluster*	2.6(1.8,3.6)	3.2(2.2,4.9)	1.6(1.1,2.6)	1.8(1.3,2.7)	3.5(2.2,6.0)	22.7(7.6,87.2)	1.7(1.0,3.4)	2.1(1.5,2.9)
Duck	1.0(0.6,1.7)	1.3(0.7,2.9)	0.8(0.5,1.6)	0.9(0.5,1.8)	1.2(0.6,3.4)	1.2(0.6,2.7)	0.9(0.2,10.6)	1.2(0.6,2.8)
Cluster*	2.6(1.9,3.7)	3.4(2.3,5.1)	1.6(1.1,2.6)	1.9(1.3,2.8)	3.7(2.4,6.3)	21.6(7.4,80.8)	1.7(1.0,3.3)	2.2(1.6,3.1)
Goose	2.8(1.2,6.9)	4.4(1.4,16.7)	1.2(0.4,4.3)	1.9(0.7,5.9)	4.3(1.1,21.8)	4.2(1.3,15.1)	0.9(0.1,26.7)	2.1(0.6,9.7)
Cluster*	2.5(1.8,3.5)	3.0(2.1,4.6)	1.6(1.0,2.6)	1.8(1.3,2.7)	3.3(2.1,5.5)	21.1(6.9,81.9)	1.7(1.0,3.4)	2.1(1.5,3.0)

^*^The local K-function estimates for the spatial clusters of H7N9 cases.

**Table 4:** Modified risks (matched Odds Ratio; 95% Confidence Intervals) of H7N9 infection associated with shortest distances to inland waters at neighborhood, community, district, and city levels.

Variable	Neighbourhood	Community	District	City
	≤1 km	1–3 km	3–5 km	5–8 km
Chicken	1.35 (1.01, 2.23)	1.96 (1.01, 3.89)	2.11 (1.00, 4.48)	2.03 (0.95, 4.3)
Cluster*	2.38 (1.71, 3.31)	1.16 (1.07, 1.27)	1.05 (1.00, 1.09)	1.03 (1.00, 1.06)
sd2river	0.997 (0.991, 1.001)	0.997 (0.991, 1.002)	0.997 (0.991, 1.002)	0.997 (0.991, 1.002)
sd2lake	0.996 (0.994, 0.998)	0.997 (0.995, 0.998)	0.997 (0.995, 0.998)	0.997 (0.994, 0.998)
Duck	1.02 (0.63, 1.64)	1.07 (0.57, 1.97)	0.80 (0.40, 1.61)	0.79 (0.39, 1.63)
Cluster*	2.44 (1.75, 3.38)	1.16 (1.06, 1.27)	1.04 (1.00, 1.07)	1.02 (1.00, 1.06)
sd2river	0.997 (0.991, 1.00)	0.997 (0.992, 1.003)	0.997 (0.991, 1.002)	0.996 (0.991, 1. 002)
sd2lake	0.996 (0.994, 0.998)	0.997 (0.995, 0.998)	0.996 (0.994, 0.998)	0.996 (0.994, 0.998)
Goose	1.78 (0.76, 4.21)	2.77 (1.01, 7.75)	2.87 (1.00, 8.79)	2.82 (0.89, 8.87)
Cluster*	2.35 (1.69, 3.28)	1.16 (1.06, 1.26)	1.04 (1.00, 1.09)	1.02 (0.99, 1.06)
sd2river	0.997 (0.991, 1.002)	0.997 (0.992, 1.002)	0.996 (0.991, 1.001)	0.996 (0.991, 1.00)
sd2lake	0.996 (0.994, 0.998)	0.997 (0.995, 0.999)	0.997 (0.996, 0.999)	0.997 (0.995, 0.999)

*The local K-function estimates for the spatial clusters of H7N9 cases.
