# Supplementary material for: Estimating Risks of Inapparent Avian Exposure for Human Infection: Avian Influenza Virus A (H7N9) in Zhejiang Province, China
Source: Sci Rep. 2017 Jan 5;7:40016. doi: 10.1038/srep40016 (PMC5214706; doi:10.1038/srep40016)
Supplement: Supplementary Information [file srep40016-s1.pdf]

Title page

Estimating Risks of Inapparent Avian Exposure for Human Infection: Avian Influenza Virus A (H7N9) in Zhejiang Province, China

Erjia Ge[1], Renjie Zhang[2], Dengkui Li[3], Xiaolin Wei[1,4], Xiaomeng Wang[5], Poh-Chin Lai[6,]*

[1] Dalla Lana School of Public Health, University of Toronto, Toronto, Canada

[2] Zhejiang Provincial Center for Disease Prevention & Control, P.R. China

[3] School of Mathematics & Statistics, Xi'an Jiaotong University, P.R. China

[4] The Jockey Club School of Public Health and Primary Care, The Chinese University of Hong Kong, Hong Kong SAR

[5] Zhejiang Provincial Center for Disease Prevention & Control, P.R. China

[6] Department of Geography, The University of Hong Kong, Hong Kong SAR.

Supplementary Tables

Table S1 Ordinal category of the shortest distances to inland waters and average poultry densities across multiple spatial scales

	Category	Range		
	Shortest distance	River(km)		Lake(km)
	shortest	0 - 23.16		0 - 93.82
	short	23.17 - 58.49		90.83 - 207.56
Inland Water	middle	58.50-223.97		207.57-336.95
	long	112.97-207.17		336.96-516.87
	very long	207.17-401.81		516.88-909.64
	Poultry density	Chicken (/km^2)	Duck (/km^2)	Goose (/km^2)
	very low	0-1.35	0-1.17	0-0.25
Neighbourhood	low	1.36-2.68	1.18-2.38	0.25-1.14
≤1km	average	2.69-2.99	2.39-2.60	1.15-1.41
	high	3-3.19	2.61-2.77	1.42 - 1.59
	very high	3.20-3.44	2.78-3.05	1.60-1.92
	very low	0-1.79	0-1.09	0-0.57
Community	low	1.80-2.70	1.10-2.28	0.58-1.14
1-3km	average	1.15-1.38	2.29-2.55	2.71-2.97
	high	1.39-1.55	2.56-2.74	2.98-3.17
	very high	1.54-1.82	2.75-3.01	3.18-3.44
	very low	0-1.52	0-1.23	0-0.64
District	low	0.65-1.15	-	1.53-2.66
3-5km	average	1.16-1.38	1.24-2.56	2.67-2.94
	high	1.39-1.55	-	2.94-3.15
	very high	1.55-180	2.57-2.98	3.16-3.43
	very low	0-1.38	0-1.17	0-0.24
City	low	0.25-1.13	1.18-2.38	1.39-2.7
5-8km	average	1.13-1.36	2.39-2.60	2.71-2.96
	high	1.37-1.54	2.61-2.77	2.97-3.16
	very high	1.55-1.79	2.78-3.05	3.17-3.42

Table S2 Interaction between shortest distances to inland waters and poultry densities in introducing H7N9 infection at community, district, and city levels

Scale	Interaction detector C = A∩B		Linear combination A+B	Graphical representation	Interpretation
	river∩chicken=0.076	>	0.046=river(0.014)+chicken(0.032)	C > A+B	⇑
	river∩goose=0.077	>	0.064=river(0.014)+goose(0.05)	C > A+B	⇑
Community	river∩duck=0.052	>	0.029=river(0.014)+duck(0.015)	C > A+B	⇑
(1-3 km)	lake∩chicken=0.086	>	0.082=lake(0.050)+chicken(0.032)	C > A+B	⇑
	lake∩goose=0.086	<	0.1=lake(0.050)+goose(0.050)	C > A, B; C < A+B	↑
	lake∩duck=0.072	>	0.065=lake(0.050)+duck(0.015)	C > A+B	⇑
	river∩chicken=0.076	>	0.046=river(0.014)+chicken(0.032)	C > A+B	⇑
	river∩goose=0.077	>	0.073=river(0.014)+goose(0.059)	C > A+B	⇑
District	river∩duck=0.041	>	0.031=river(0.014)+duck(0.017)	C > A+B	⇑
(3-5 km)	lake∩chicken=0.086	>	0.082=lake(0.050)+chicken(0.032)	C > A+B	⇑
	lake∩goose=0.076	<	0.109=lake(0.050)+goose(0.059)	C > A, B; C < A+B	↑
	lake∩duck=0.073	>	0.067=lake(0.050)+duck(0.017)	C > A+B	⇑
	river∩chicken=0.084	>	0.072=river(0.014)+chicken(0.058)	C > A+B	⇑
	river∩goose=0.057	>	0.05=river(0.014)+goose(0.036)	C > A+B	⇑
City	river∩duck=0.058	>	0.044=river(0.014)+duck(0.030)	C > A+B	⇑
(5-8 km)	lake∩chicken=0.101	<	0.108=lake(0.050)+chicken(0.058)	C > A, B; C < A+B	↑
	lake∩goose=0.073	<	0.086=lake(0.050)+goose(0.036)	C > A, B; C < A+B	↑
	lake∩duck=0.093	>	0.080=lake(0.050)+duck(0.030)	C > A+B	⇑

Note: A and B indicate inland waters and poultry densities respectively; A ⇑ B denotes nonlinear enhancement of A and B when C > A+B; A ↑ B denotes A and B enhance each other when C > A, B.

Table S3 Estimated risks (matched Odds Ratio; 95% Confidence Intervals) of H7N9 infection associated with poultry species and densities at community, district, and city levels

		All population	Gender		Age (years old)		Occupation		
			Male	Female	15-59	≥60	Farmer	Worker	Others
	Variable	142 cases<br>599 controls	93 cases<br>369 controls	49 cases<br>190 controls	73 cases<br>281 controls	66 cases<br>261 controls	59 cases<br>235 controls	15 cases<br>96 controls	68 cases<br>228 controls
Community (1-3 km)	Chicken	2.9(1.4,6.2)	4.2(1.6,12.8)	1.6(0.6,5.3)	1.7(1.0,4.3)	10.4(2.6,47.2)	2.48(1.0,7.4)	1.5(0.1,28.1)	4.3(1.2,19.9)
	Cluster*	1.2(1.1,1.3)	1.2(1.1,1.3)	1.1(1.0,1.3)	1.2(1.0,1.4)	1.2(1.1,1.3)	1.8(1.2,2.8)	1.2(1.0,1.5)	1.2(1.1. 1.3)
	Duck	1.2(0.6,2.3)	1.4(0.6,4.2)	0.8(0.3,2.0)	0.8(0.4,1.7)	1.2(0.6,3.4)	1.2(0.5,3.1)	0.7(0.1,14.9)	1.2(0.5,3.7)
	Cluster*	1.2(1.1,1.3)	1.2(1.1,1.4)	1.1(1.0,1.3)	1.2(1.1,1.4)	1.3(1.1,1.4)	1.9(1.3,2.9)	1.2(1.0,1.5)	1.2(1.1,1.3)
	Goose	5.0(1.8,3.5)	7.4(2.0,31.5)	1.7(0.4,8.5)	2.0(0.7,7.2)	19.9(3.6,138.3)	8.3(2.1,38.6)	0.5(0.0,28.1)	2.8(0.6,17.4)
	Cluster*	1.2(1.1,1.3)	1.2(1.1,1.3)	1.1(1.0,1.3)	1.2(1.1,1.3)	1.2(1.1,1.3)	1.6(1.1,2.5)	1.2(1.0,1.5)	1.06(1.02,1.1)
District (3-5 km)	Chicken	3.5(1.6,7.7)	4.9(1.8,15.0)	1.8(0.6,6.4)	1.7(0.7,4.7)	13.1(3.3,59.7)	2.9(1.0,9.4)	1.8(0.2,36.8)	4.8(1.3,21.7)
	Cluster*	1.1(1.0,1.1)	1.1(1.05,1.5)	1.03(1.0,1.1)	1.06(1.0,1.4)	1.09(1.04,1.15)	1.4(1.1,9.4)	1.08(1.0,1.5)	1.08(1.03,1.1)
	Duck	1.1(0.5,2.2)	1.4(0.5,4.7)	0.7(0.3,1.8)	0.7(0.3,1.5)	5.2(1.1,29.3)	1.0(0.4,2.9)	0.8(0.1,25.2)	0.9(0.3,3.4)
	Cluster*	1.1(1.0,1.1)	1.1(1.05,1.16)	1.03(1.0,1.1)	1.06(1.0,1.13)	1.1(1.06,1.2)	1.5(1.1,1.9)	1.08(1.0,1.2)	1.07(1.02,1.12)
	Goose	5.9(2.0,17.7)	8.6(2.2,38.4)	2.0(0.5,10.7)	2.1(0.6,8.2)	25.9(4.4,188.4)	9.1(2.1,44.6)	0.8(0.2,48.6)	3.4(0.6,23.8)
	Cluster*	1.1(1.0,1.1)	1.08(1.04,1.14)	1.03(1.0,1.1)	1.06(1.0,1.1)	1.07(1.02,1.1)	1.3(1.0,1.7)	1.1(1.0,1.2)	1.2(1.1,1.3)
City (5-8 km)	Chicken	3.3(1.5,7.3)	4.4(1.6,13.1)	1.8(0.6,6.9)	1.9(1.0,5.2)	9.4(2.5,39.9)	2.4(1.0,7.9)	3.3(0.3,77.1)	3.5(1.0,14.9)
	Cluster*	1.06(1.02,1.09)	1.07(1.04,1.1)	1.02(1.0,1.1)	1.06(1.02,1.1)	1.06(1.02,1.1)	1.3(1.1,1.5)	1.1(1.0,1.2)	1.05(1.01,1.08)
	Duck	1.1(0.5,2.4)	1.7(0.6,6.1)	0.7(0.3,1.8)	0.8(0.4,1.7)	4.9(1.0,29.5)	1.0(0.4,2.7)	3.2(0.1,57.7)	0.8(0.3,2.8)
	Cluster*	1.06(1.03,1.09)	1.08(1.05,1.12)	1.01(1.0,1.1)	1.06(1.01,1.1)	1.08(1.04,1.1)	1.3(1.1,1.5)	1.1(1.0,1.2)	1.04(1.01,1.08)
	Goose	5.8(1.9,17.9)	7.5(1.9,33.8)	2.4(0.5,13.7)	2.2(0.6,8.9)	22.6(3.9,161.8)	7.1(1.7,35.2)	1.7(0.1,135.5)	2.9(0.5,20.4)
	Cluster*	1.05(1.02,1.08)	1.06(1.03,1.1)	1.01(1.0,1.07)	1.05(1.0,1.1)	1.05(1.0,1.1)	1.6(1.1,2.5)	1.1(1.0,1.2)	1.04(1.0,1.1)

* The local K-function estimates for the spatial clusters of H7N9 cases.
